# Development of High-Throughput Serum Bactericidal Assays for *Bordetella pertussis* to Evaluate BPZE1

**DOI:** 10.3390/vaccines14060492

**Published:** 2026-05-30

**Authors:** Peter Goldstein, Tania Gensale, Shannon Harris, Tina M. Green, Stephanie Noviello, Keith Rubin, Camille Locht, Breeze Cavell, Andrew Gorringe, Luc Gagnon

**Affiliations:** 1 ILiAD Biotechnologies, Inc., Weston, FL 33331, USA; 2 Department of Biomedical Engineering, University of Miami, Miami, FL 33146, USA; 3 IQVIA Laboratories, Laval, QC H7V 3S8, Canada; 4 CNRS, Inserm, CHU Lille, Institut Pasteur de Lille, U1019-UMR9017–CIIL—Centre for Infection and Immunity of Lille, University of Lille, F-59000 Lille, France; 5 UK Health Security Agency, Porton Down SP4 0JG, UK

**Keywords:** pertussis, whooping cough, BPZE1, bactericidal titers, pertactin, complement-mediated, SBA

## Abstract

Background/Objectives: Pertussis, caused by *Bordetella pertussis*, remains a global health problem, despite high vaccine coverage. In countries with high acellular pertussis vaccine (aPV) coverage, pertactin-negative *B. pertussis* strains emerged due to vaccine pressure on the sole bactericidal target of aPVs. In contrast, the live attenuated intranasal vaccine BPZE1 induces bactericidal antibodies to multiple antigenic targets that kill pertactin-positive and pertactin-negative *B. pertussis* strains. Here, we developed two high-throughput human complement-mediated serum bactericidal assays (SBA) using clinical samples to demonstrate bactericidal activity against *B. pertussis*. Methods: Assay accuracy, precision, linearity, range and robustness of the SBAs against pertactin-positive and pertactin-negative *B. pertussis* strain B1917 were determined using a panel of commercial and clinical trial samples. The assay was used to analyze a cohort of BPZE1 and tetanus–diphtheria–acellular pertussis (Tdap) vaccinee samples at baseline and 28 days post-vaccination from a phase 2b clinical trial. Results: Inter- and intra-assay variability of both assays had coefficients of variation for repeatability < 20% and for intermediate precision of <30%. The assays measured titers ranging from ~8 to ~20,000 and showed high linearity (R^2^ > 0.98) between bactericidal titers and serum dilutions. On clinical samples, BPZE1 induced similar bactericidal activity as Tdap against pertactin-positive *B. pertussis*, despite inducing lower anti-aP antigen IgG concentrations than Tdap. Additionally, BPZE1 induced serum bactericidal activity against pertactin-negative *B. pertussis*, while Tdap did not. Conclusions: High-throughput SBAs were developed and qualified against pertactin-positive and pertactin-negative *B. pertussis*, enabling measurement of 120 samples per day per analyst. These assays will support clinical development of next-generation pertussis vaccines, including BPZE1.

## 1. Introduction

*Bordetella pertussis*, an aerobic Gram-negative bacterium, is the main causative agent of whooping cough or pertussis, a highly contagious respiratory disease and one of the least controlled vaccine-preventable childhood diseases [1]. Pertussis is considered endemic throughout the world, with 1–5% of the population experiencing sub-clinical *B. pertussis* colonization each year [2,3] representing a large bacterial reservoir to spread to those without adequate immunity, including the young, elderly and immunocompromised. After the SARS-CoV-2 pandemic, large outbreaks of pertussis were reported around the world. In the US and UK cases tripled from 2023 to 2024 with >40,000 cases reported in the US and >15,000 in the UK [4]. In the EU, cases increased by more than 10 times that of any year in the prior decade in many countries, including France, the Czech Republic, and Finland, with similar trends observed in Australia [4]. In China over 130,000 cases were reported between February and April 2024 alone, more than any year since 1985 [5].

Two types of injected pertussis vaccines are currently approved for use in the world, acellular pertussis vaccines (aPVs) and whole-cell pertussis vaccines (wPV). aPVs consist of one to five of the following protein antigens, pertussis toxin (PT), filamentous hemagglutinin (FHA), pertactin (PRN), and serotypes 2 and 3 fimbriae (FIM2/3), adsorbed onto aluminum hydroxide adjuvant, while wPVs comprise a suspension of inactivated *B. pertussis*. In the 1990s and early 2000s, high-income countries switched from wPVs to aPVs [6], due to concerns of vaccine side effects from wPVs. Soon after the transition to aPVs, pertussis frequencies increased, although rates of severe disease remained stable [2,7].

One reason for this increase was the fact that injected aPVs do not prevent infection (i.e., nasopharyngeal colonization), and consequently do not prevent human-to-human transmission of *B. pertussis* [3]. Additionally, aPV-induced immunity wanes faster than immunity induced by wPVs [8,9,10,11]. Finally, aPV pressure has driven evolution of new strains, capable of evading vaccine-induced immunity [12,13,14]. In particular, strains lacking PRN (PRN−), the only antigen present in aPVs shown to induce bactericidal antibodies [15], have emerged, representing up to 80% of the circulating strains in countries using aPVs [14,16,17]. These observations suggest that, although aPVs do not prevent infection, they induce enough PRN-specific bactericidal activity to enable PRN-deficient escape mutants to pervade, and that serum bactericidal activity against *B. pertussis* may indicate occurrence of bactericidal activity in the lung or respiratory mucosa [18]. As *B. pertussis* is a strictly mucosal pathogen, it may be hypothesized that bactericidal antibodies exudate from the serum to mucosal sites [19,20] to initiate the classical antibody-mediated complement killing pathway. Since levels of antibodies in mucosal fluids are typically much less than those found in serum, demonstrating mucosal bactericidal activity in vitro using nasal fluid samples is still elusive. Increases in complement components, including the membrane attack complex C5-b9, have been shown to become elevated in mucosal inflammatory conditions [21,22] further supporting the potential for this mechanism. However, currently the only specimens available to demonstrate complement-mediated bactericidal activity are from blood.

Given the importance of bactericidal antibodies against *B. pertussis*, as suggested by the emergence of PRN-deficient escape mutants, demonstrating the ability of human serum to induce bactericidal antibodies to both pertactin-positive (PRN+) and PRN− strains after vaccination may provide support for the efficacy of novel pertussis vaccines against the prevalent circulating strains of *B. pertussis*.

BPZE1 is a live attenuated intranasal pertussis vaccine candidate, demonstrated to be safe and capable of inducing mucosal and serum antibodies in six clinical trials [23,24,25,26,27]. BPZE1 has demonstrated protection against *B. pertussis* infection in baboons [28] and humans in challenge studies [23,27].

In a Phase 2b clinical study comparing BPZE1 with tetanus-reduced diphtheria–acellular pertussis vaccine (Tdap), serum bactericidal antibody responses were evaluated in a small cohort of 30 participants, utilizing a tilt-plate serum bactericidal assay (SBA) [15]. In this cohort, BPZE1 demonstrated the ability to induce complement-mediated bactericidal activity against both PRN+ and PRN− variants of *B. pertussis* strain B1917, while Tdap only induced bactericidal activity against the PRN+ strain [27].

To monitor bactericidal activities in large studies, we developed and qualified robust high-throughput SBAs against PRN+ and PRN− B1917 using a clear agar-overlay method, which enabled automated imaging of multiple samples tested in a 96-well plate format. This method enables handling of far more samples per day than the tilt-plate method, reducing the amount of reagents, incubator space, and hands-on time of analysts. We then re-tested the set of serum samples previously analyzed [27] to ensure the assay performed similarly to the tilt-plate method to support further development.

## 2. Materials and Methods

### 2.1. Strains, Complement, Critical Reagents

*B. pertussis* strain B1917 [29], a representative circulating PRN+ strain, was kindly provided by the RIVM (Bilthoven, The Netherlands). The PRN− *B. pertussis* B1917 derivative was described earlier [15]. Pooled IgG/IgM-depleted human serum (Code# 34010) was obtained from Pel-Freeze Biologicals (Rogers, AR, USA). The WHO 1st International Standard Antiserum (06/140) was obtained from the National Institute for Biological Standards and Control (NIBSC, Hertfordshire, UK) and used as a positive control.

### 2.2. Vaccines

Two vaccines were used in the clinical trial [27]: BPZE1 (ILiAD Biotechnologies, Inc., Weston, FL, USA) and Tdap, containing PT, FHA, and PRN (Boostrix^TM^, GlaxoSmithKline, London, UK).

### 2.3. Serum Samples

For method development and qualification, commercial serum samples (Catalog #: HUMANSRM-0101228, BioIVT, Woodbury, NY, USA) with a range of bactericidal titers (0 to ~20,000) were evaluated, along with clinical trial samples [27].

Serum samples obtained at baseline and 28 days after vaccination during the study [27] were analyzed using an electrochemiluminescent assay to quantify serum IgG antibodies against PRN (Meso Scale Diagnostics, Rockville, MD, USA) to evaluate the relation of SBA titer to anti-PRN IgG concentration.

### 2.4. Agar-Overlay SBA with PRN+ and PRN− B. pertussis

**Figure 1:**
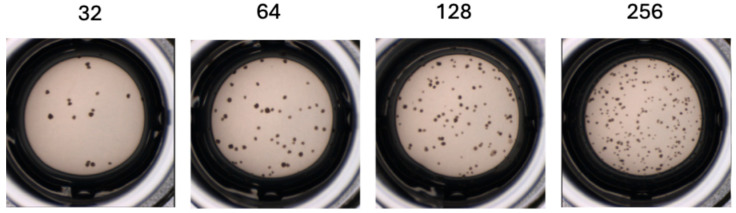
Example image of agar-overlay SBA showing an increase in CFUs (black spots) as the test serum concentration decreased via 2-fold serial dilutions. Reciprocal dilution values listed above.

The high-throughput SBA was developed to evaluate the functional activity of vaccine-induced antibodies in the presence of human complement (IgG/IgM-depleted) to kill both PRN+ and PRN− *B. pertussis* strain B1917. Bactericidal measurements were conducted on thawed serum samples, which were subsequently heat-inactivated at 56 ± 2 °C for 30 ± 5 min. Two-fold serial dilutions of the heat-inactivated serum samples from 1:8 to 1:4096 were prepared in 96-well plates yielding a final volume of 25 ul per well. A standardized target of ~150–400 colony-forming units (CFUs) of *B. pertussis* strain B1917 or its PRN− derivative together with purified IgG/IgM-depleted human complement (Pel-Freeze, Rogers, AR, USA) at a final percentage of 15% were then added, and the plates were incubated for 120 ± 5 min at 37 ± 2 °C with 5% CO_2_, shaking and 65% humidity to allow binding of the antibodies to the bacteria and induction of the complement cascade resulting in bacterial cell death. The complement–antibody–bacteria mixture was then overlaid with 100 ul of an agar preparation of Bordet–Gengou media modified without blood products containing 0.9% agar (Becton Dickenson, Ontario, Canada) prewarmed to 48 °C, and incubated for 4 days at the same conditions above. The lack of blood products enables the automated image analysis equipment to count all colonies growing throughout the media in each well of the 96-well plate as the agar is clear rather than dark red (Figure 1).

**Figure 2:**
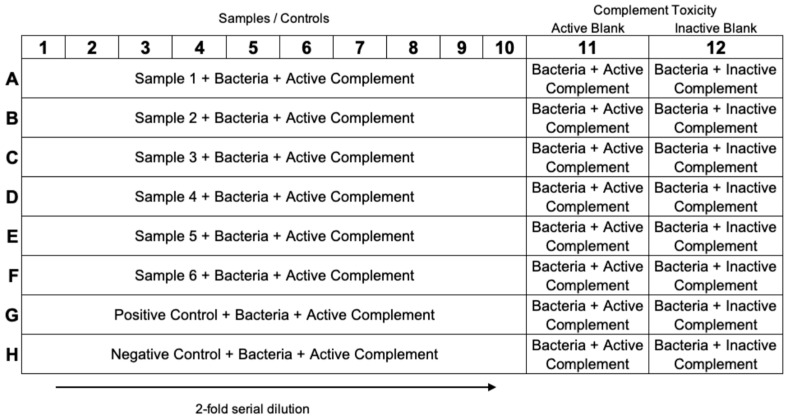
Plate layout of the SBA. Individual samples are tested on rows A–F and two-fold serially diluted from column 1 to column 10. Rows G and F contain pre-defined positive and negative controls diluted in the same manner. The CFU count in each sample is divided by the GMT of the CFU counts of column 11 to generate the bactericidal titer. Column 12 reports the average CFUs of bacteria + heat-inactivated complement and is used to assess intrinsic complement toxicity.

The bacterial colonies were then imaged with a Zeiss AxioLab, and counted using an automated image analysis system (AxioVision version 4.8.2 with ICARUS version 1.2.3). The bactericidal titer for each serum sample was calculated by dividing the geometric mean CFU count of the 8 samples in the control wells containing active complement + bacteria (Figure 2, column 11) by the CFUs in the wells with dilutions of heat-inactivated human serum samples. The reciprocal serum dilution generating 50% killing using a continuous titer calculation was reported as the SBA titer.

#### 2.4.1. High-Throughput Assay Workflow

Each assay run is designed to process up to twenty plates, totaling 120 test samples in addition to quality controls (six samples and two quality controls per plate). With each plate containing 96 wells, a single assay run involves counting colonies in 1920 wells.

Automated image acquisition and colony enumeration for all plates in one run are completed in approximately three hours. This automated workflow significantly reduces operator involvement and analysis time compared to conventional manual counting methods. Furthermore, this approach reduces operator-dependent bias, enhancing consistency and reproducibility of colony enumeration.

#### 2.4.2. Colony Counting: Automation and Supervision

Colony enumeration is performed using a semi-automated image analysis workflow. Test plates are loaded into the automated imaging system, and images of each well are acquired using the Zeiss AxioLab automated system, where the colonies appear black on a white background (Figure 1). Identification and counting of colonies are conducted automatically using either AxioVision v4.8.2 or ICARUS v1.2.3 software, based on pre-defined segmentation parameters such as colony size/morphology. Hence, the software is trained to identify and classify colonies based on pre-defined morphological characteristics, including size and shape.

To ensure the accuracy and robustness of the data, automated counts can be subject to manual verification (visual verification of the image of each well). If colony-forming unit (CFU) count adjustment is required, a manual counting tool is used to add missing or remove incorrect CFU counts for a given well. This is especially important when colony clustering, irregular morphology, or background artifacts could impact segmentation performance. This approach allows the assay to maintain high throughput without compromising data quality.

#### 2.4.3. Image Analysis Software and Alternatives

Image analysis is performed using the AxioLab, which integrates image acquisition and colony counting in conjunction with the AxioVision version 4.8.2 or ICARUS version 1.2.3 software programs.

Comparable data acquisition can also be achieved using alternative automated imaging systems such as the Cytation 7. This alternative provides flexibility and accessibility and may be suitable depending on laboratory infrastructure and user expertise.

### 2.5. Plate Layout

The plate layout allowed for simultaneous testing of 6 samples, with ten 2-fold serial dilutions (Figure 2, in rows A through F, columns 1–10). Columns 11 and 12 served as controls. Column 11 contained bacteria and active complement and is used to determine the bactericidal titer of each sample, while column 12 contained bacteria + inactive complement and is used to assess intrinsic bactericidal activity of the complement source. Rows G and H served as additional positive and negative controls, respectively, to ensure the assay performs consistently. If either of the controls failed, the entire plate was re-tested. Additionally, the 1st International Pertussis Standard [30] 06-140 was included as a sample in precision tests, and was included as one sample in every batch of plates tested during clinical testing (~20 plates per batch) for trending.

### 2.6. Assay Qualification

Assay qualification studies were performed to demonstrate precision, dilution linearity, limits of quantitation, specificity, matrix interference, freeze–thaw stability, and robustness using a panel of 20 serum samples spanning the assay range. Each assay plate included two quality control human serum samples (one mid-positive control and one negative control). The mid-positive control was composed of commercial human serum samples (BioIVT, Woodbury, NY, USA) and the negative control was composed of commercial IgG/IgM-depleted human serum (Pel-Freeze Biologicals, Rogers, AR, USA). The serum samples (individual and pooled heat-inactivated samples) were stored at −80 °C (−60 to −90 °C) in aliquots and thawed prior to use on the day of testing by placing them at room temperature (RT) until completely thawed.

#### 2.6.1. Precision

Precision was determined by evaluating repeatability (intra-assay variation) and the intermediate precision (intra- and inter-assay variation) using a panel of 20 individual and pooled human serum samples with titers covering the analytical range of the assay (high, mid, low and negative). The WHO International Standard Pertussis Antiserum (Human) 1st IS NIBSC 06/140 was used as a sample for the precision assessment in order to calibrate the assay. Samples were assessed in duplicate (independent preparations loaded on the same assay plate) over 3 days by 2 analysts (in parallel) to generate a total of 12 measurements per sample.

Goemetric mean titers (GMTs) and a coefficient of variation (CV) for each sample were calculated using the formulas:

(1) $${GMT}_{\bar{y}}=\;\sqrt[{n}]{y_{1\;}y_{2}y_{3}{\ldots y}_{n}}\;$$

and

(2) $$CV(\%)=100\%\cdot \sqrt{10^{\ln\left(10\right)\sigma^{2}}-1}$$

where:

- y is an obtained value (titer);
- n is the number of values obtained;
- σ^2^ is the overall variability of the values in log10 scale (calculated for each parameter being assessed).

Intra-assay CV, overall CV, lower limit of precision (LLP) and upper limit of precision (ULP) were determined. The latter corresponded respectively to the lowest and highest GMT that could be measured with acceptable precision.

#### 2.6.2. Dilutional Linearity/Relative Accuracy

Dilutional linearity testing was performed on 5 positive human serum samples diluted to target titers covering the analytical range of the assay (high, mid and low). For each dilution and corresponding neat sample, 3 independent preparations were produced. The dilutional linearity (and relative accuracy) was calculated as the ratio between the geometric mean of the triplicate measured values (multiplied by the corresponding dilution factor) to the expected value. Each independent preparation was tested at the optimal starting dilution in parallel with the corresponding neat sample.

#### 2.6.3. Lower and Upper Limits of Quantitation (LLOQ and ULOQ)

LLOQ and ULOQ were determined based on the precision and linearity profiles obtained with the panel of human serum samples used to assess the precision and the dilutional linearity. The maximum lower limit of either precision or linearity was selected to be the LLOQ, and the minimum upper limit of precision or linearity was selected to be the ULOQ.

#### 2.6.4. Specificity

Five positive human serum samples (15 ul) with titers covering the analytical range were competed against an equal volume of 25.0 µg/mL of *B. pertussis* whole-cell extract (WCE) (Item # BA120VS8, Serion Immunologics, Würzburg, Germany) as a homologous antigen, while 25.0 ug/mL of diphtheria toxoid or tetanus toxoid (products DAG2689, DAG2692, Creative Diagnostics, Shirley, NY, USA) were used as heterologous antigens. The serum samples were pre-treated for 60 min at RT with an equal volume of homologous or heterologous competitor or without competitor (mock). The competed samples and the mock treated samples were then tested in the same assay run.

Specificity was determined by calculating the change in the observed titer of each sample pre-treated with competitor compared to the corresponding untreated sample using the following formula.

%inhibition = (1 − (titer of competed sample/titer of mock sample)) × 100(3)

#### 2.6.5. Matrix Interference

Matrix interference was evaluated according to the CLSI-C56A guidelines [31] by testing 6 human serum samples with titers covering the analytical range, including 1 negative sample. Briefly, serum samples were diluted with assay diluent (Gibco^TM^ HBSS [ThermoFisher, Canada] + 1% BSA [MilliporeSigma Canada Ltd., Ontario, Canada]) or treated with high levels of interferent (250 mg/dL hemoglobin [BioIVT, Woodvury, NY, USA], 250 mg/dL lipid [Item # I141, MilliporeSigma Canada Ltd., Ontario, Canada] or 16 mg/dL bilirubin [Item # 201102, Calbiochem®, MilliporeSigma Canada Ltd., Ontario, Canada]). Before testing in the SBA, samples treated with hemoglobin were incubated for 60 min on ice while mock treated samples and samples treated with lipid and bilirubin were incubated for 60 min at RT. Untreated and corresponding treated samples spiked with interferent were measured on the same plate.

The percentage recovery was determined by comparing the titer obtained for the treated sample compared to the titer of the corresponding untreated sample using the following formula:

%Recovery = (titer of treated sample/titer of untreated sample) × 100(4)

#### 2.6.6. Freeze/Thaw and Bench Top Stability

A set of six human serum samples with titers covering the analytical range of the assay (high, mid, and low) were divided into 4 aliquots and stored frozen at −80 °C. One aliquot was thawed once on the day of testing and was considered as the reference (1 freeze/thaw cycle on the day of testing). The second aliquot was thawed once on the day before testing and was used to test bench top stability. The 2 remaining aliquots underwent 5 and 10 freeze/thaw cycles where each sample was frozen at −80 °C for at least 12 h before thawing at RT (15 to 30 °C) for 4 ± 1 h. The stability sample aliquots were frozen again at −80 °C for at least 12 h before the next freeze/thaw cycle. The last freeze/thaw cycle was completed on the day of the run, and samples were loaded in the assay plate when completely thawed.

For each condition a geometric mean ratio (GMR) of the measured value to the reference value was calculated using the following formula:

(5) $${GMR}_{\bar{y}}=\;\sqrt[{n}]{y_{1\;}y_{2}y_{3}{\ldots y}_{n}}\;$$

where:

- y is an obtained value (ratio of measured value to reference condition);
- n is the number of values obtained.

#### 2.6.7. Robustness

To understand the plating time effect corresponding to handling 20 plates in one assay run, the maximum batch size for one operator in a day, a set of 7 or 8 human sera covering the analytical range for the PRN+ and PRN− SBAs, respectively, were tested on 20 plates by one analyst. The same set of samples and controls was tested on all plates of a run at the same starting dilution, which included 4 positive serum samples, 1 positive control, 1 negative sample and 1 negative control. The GMT of each sample’s results on the last 5 plates was considered its reference titer. The ratios of each sample titer to its reference titer on each of the 20 plates were determined, and the GMR of each set of ratios for each plate was calculated.

To assess the stability of the readings over time, additional image acquisitions were performed 6 and 10 days following the end of the first reading. Forty samples from one of the precision runs were used and assigned a value T0. After initial analysis the plates were sealed and stored at 4 °C for the additional incubation time until being read again.

To analyze extended reading time, the ratios of 40 analyzed samples to their reference condition (T0) were evaluated. Each sample was tested in duplicate during two runs of the precision assessment. The GMRs were calculated by comparing the extended condition to the reference condition (T0) (Supplementary Tables S9 and S10).

#### 2.6.8. Quality Control Range Determination

To monitor the consistency of the assay results over time, a mid-positive quality control (QC) sample and a negative QC sample previously determined to have a bactericidal titer < 8 were prepared by pooling human serum samples. Both QCs were run in parallel with the measurement of unknown samples to evaluate whether the SBA operated within pre-defined target acceptance limits. Assay performance and trend data were compiled. For the PRN+ SBA, 101 data points were compared from two analysts testing 13 separate SBA runs over 6 calendar weeks. For the PRN− SBA, 115 data points from three analysts testing 14 separate assay runs over 5 weeks were compared. The GMT and lower and upper limits of the QC samples were calculated from these results. All calculations were performed on the log10 transformed titers, then back transformed for use in the assay. The range for the mid-positive QC set used ±3SD for the PRN+ sample set, and ±2SD for the PRN− sample set. Although the PRN− assay data was accumulated with a similar number of runs and over a similar timeframe as the PRN+ assay, data were generated by three analysts, which enhances the robustness/variability of the control limits compared to PRN+ SBA resulting in a range based on ±2SD rather than ±3SD.

PRN+ QC Range = 10 ^mean±3SD^ (6)

PRN− QC Range = 10 ^mean±2SD^ (7)

#### 2.6.9. Bacteria Control with Active Human Complement Range Determination

The bacteria with active human complement control is an important measure, as it is used to calculate the % bactericidal activity of each sample tested on each plate and can be used to monitor assay performance. This control consists of the bacterial preparation in assay diluent and active complement (loaded 8 times per plate in column 11).

#### 2.6.10. Statistical Methods

Paired or independent *t*-tests on log transformed data were used to assess bactericidal titers and anti-PRN IgG concentrations within or between treatment groups using Graphpad Prism 10. One-, two-, and three-way ANOVA analysis in SAS v9.4 was used to assess CV associated with precision measurements on log transformed titers, evaluating factors of operator, day, and sample. For precision assessment, samples with at least 6 out of 12 (i.e., 50%) valid results (reportable titers) obtained were included in the statistical analysis.

#### 2.6.11. Quality Assurance and Regulatory Compliance

All aspects of the qualification studies were performed in accordance with International Conference for Harmonisation—Good Clinical Practices (ICH-GCLP) guidelines, and internal procedures with oversight from the Quality Assurance department of IQVIA Laboratories Canada.

#### 2.6.12. Clinical Sample Analysis

The qualified high-throughput PRN+ and PRN− SBAs were used to analyze samples from the BPZE1 Phase 2b study [27]. Serum samples from baseline and day 29 post-vaccination from 30 participants, either BPZE1- or Tdap-vaccinated, as described [27], were analyzed. GMTs were calculated for each vaccine group and statistical comparisons between baseline and/or day-29 titers for PRN+ SBA and for PRN− SBA were conducted using paired and unpaired *t*-tests on log transformed titers.

## 3. Results

### 3.1. PRN+ and PRN− B. pertussis SBA Qualification

#### 3.1.1. Precision

**Figure 3:**
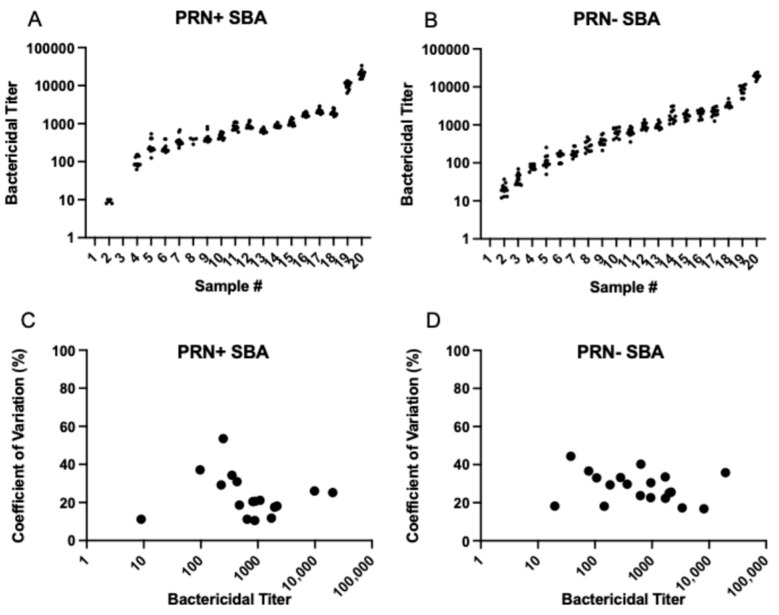
Distribution of precision panel samples (**A**,**B**) and coefficients of variation for PRN+ SBA and PRN− SBA (**C**,**D**). Panels (**A**,**B**): the reportable bactericidal titers are plotted for each sample. Sample 1 was <8 for all replicates and thus did not have reportable titers for either SBA. Sample 3 did not have reportable bactericidal titers for the PRN+ SBA. Panels (**C**,**D**): the CV of each reportable sample’s replicates are plotted on the y-axis with bactericidal titers plotted on the x-axis.

For the Repeatability and Reproducibility (R&R) analysis, 193 and 217 datapoints were evaluated for the PRN+ and PRN− SBAs respectively (Figure 3, Supplementary Tables S1 and S2). For the PRN+ SBA, the intra-assay precision CV of samples tested on the same plate by the same operator was determined to be 10.8%. The CV of day-to-day variability with the same operator and CV of inter-operator variability were 21.7% and 25%, respectively. The overall CV associated with the intermediate precision was 25.5% (Table 1). Based on a three-way ANOVA, the titers associated with the LLP and ULP were 9 (based on Sample 2) and 20,515 (based on Sample 20), respectively. Sample 2 was used to establish the LLP for this early-stage qualification despite having 5 of 12 reportable titers. Sample 1 remained below the limit of quantitation for all samples tested (<8) and thus was unable to support the LLP. The LLP will be further assessed during assay validation with a more complete panel of samples.

For the PRN− SBA, the intra-assay precision CV of samples tested on the same plate by the same operator was 19.3%. The CV of day-to-day variability with the same operator and CV of inter-operator variability were 28.4% and 27.8%, respectively. As seen in the PRN+ SBA, Sample 1 also remained below the limit of quantitation for all samples tested in the PRN− SBA (<8) and thus was unable to support the LLP. The overall CV of intermediate precision was 28.4% (Table 1). Based on a three-way ANOVA, the LLP and ULP were 20 (based on Sample 4) and 19,387 (based on Sample 18), respectively.

#### 3.1.2. Dilutional Linearity and Relative Accuracy

For the PRN+ SBA, four dilutions (1/2, 1/4, 1/8 and 1/16) for one sample, five dilutions (1/2, 1/4, 1/8, 1/16 and 1/32) for three samples and six dilutions (1/2, 1/4, 1/8, 1/16, 1/32 and 1/64) for one sample were prepared in human serum previously shown to have no bactericidal activity, so that it would not affect the results. For the PRN− SBA, five dilutions (1/2, 1/4, 1/8, 1/16 and 1/32) for two samples, six dilutions (1/2, 1/4, 1/8, 1/16, 1/32 and 1/64) for two samples, and seven dilutions (1/2, 1/4, 1/8, 1/16, 1/32, 1/64 and 1/128) for one sample were prepared in assay diluent.

**Figure 4:**
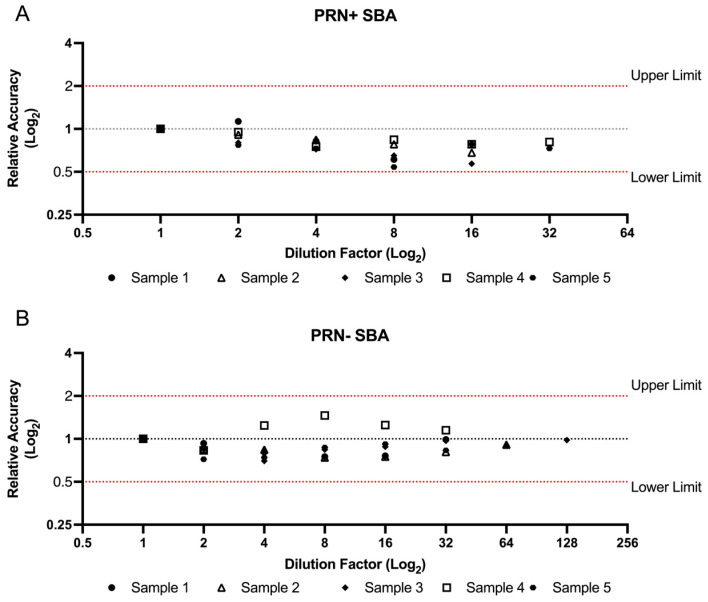
Relative accuracy for (**A**) PRN+ SBA and (**B**) PRN− SBA. The ratios of measured values vs. theoretical values for 5 samples were plotted on the y-axis vs. dilution factor ranging from 1/2 to 1/32 on the x-axis for the PRN+ SBA, and 1/2 to 1/128 for the PRN− SBA. Red dashed lines represent the upper and lower limits of the target acceptance criteria from 0.5 to 2.0.

**Figure 5:**
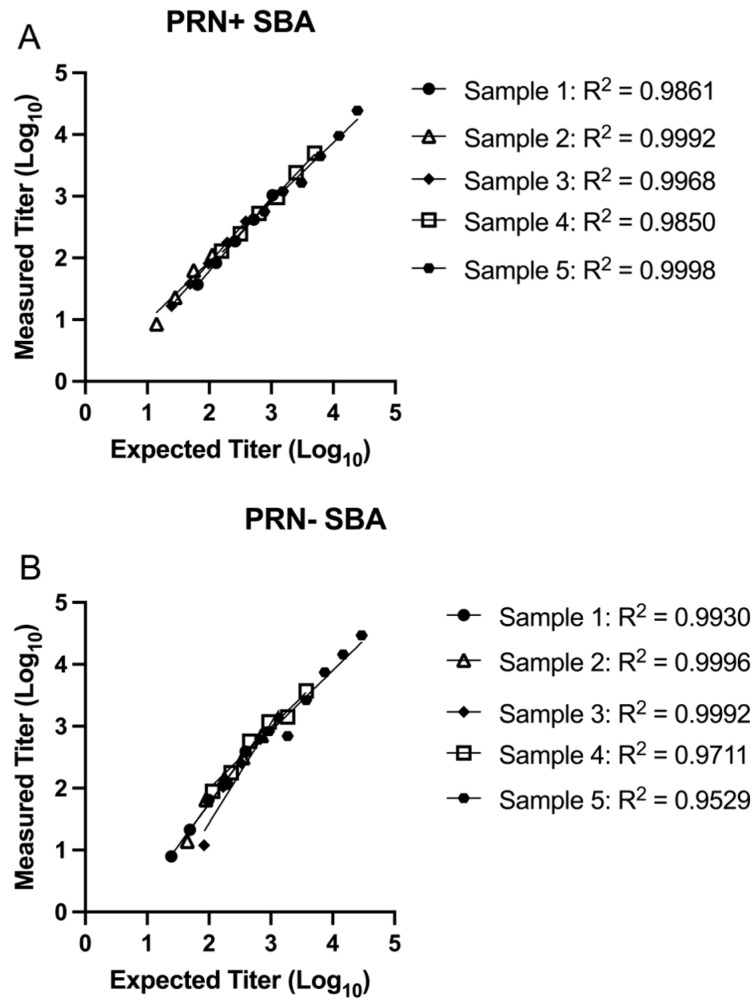
Linear relationship between expected and measured titers across multiple dilutions for (**A**) PRN+ SBA and (**B**) PRN− SBA. Linear regression of log_10_ measured titer vs. log_10_ of the expected titer at each dilution demonstrated both assays are linear in nature spanning the range of the assay with each set of five samples demonstrating R^2^ value > 0.95.

For the PRN+ SBA, the relative accuracies for all samples fell within the target acceptance limits of [0.5–2.0] (Figure 4A) from a GMT of 9 up to a maximum GMT of 24,692, the LLL and ULL respectively. For each sample the log_10_ transformed measured titers were plotted against log_10_ of the expected titers for each dilution of each sample as shown (Figure 5A). A fitted regression line was calculated using the least-squares method for each sample. All five serum samples individually had an R^2^ ≥ 0.95 and slope within the range of −1.33 to −0.67, demonstrating acceptable dilutional linearity across the range of the assay.

For the PRN− SBA, the initial experiments included samples diluted in negative matrix and met the target criteria for relative accuracy; however, dilutional linearity could not be demonstrated at the lower end of the assay (titers < 64), in particular for low titer samples. Two serum samples were then tested in parallel in either negative matrix or assay diluent starting at a 1/8 dilution. Both samples demonstrated dilutional linearity in assay diluent, while only one demonstrated dilutional linearity in negative serum matrix. Subsequently, the studies were repeated using assay diluent to dilute the samples rather than low-titer human serum. Five to seven dilutions were prepared in assay diluent, with three independent preparations each. All five serum samples had an R^2^ ≥ 0.95 and slope within the range of −1.33 to −0.67, demonstrating acceptable dilutional linearity (Figure 4B). The relative accuracies for 100% of the samples fell within the target acceptance limits of [0.5–2.0] (Figure 4B) from a GMT of 9 up to a maximum GMT of 18,907, which were assigned as the LLL and ULL for PRN− SBA.

#### 3.1.3. Lower and Upper Limits of Quantification (LLOQ and ULOQ)

For the PRN+ SBA, the LLOQ was determined to be 9, based on the lower limit of precision and the lower limit of linearity, while the ULOQ was determined to be 20,515, based on the lesser value of the upper limits of precision and linearity.

For the PRN− SBA, the LLOQ was determined to be 20, based on the lower limit of precision and the lower limit of linearity, while the ULOQ was determined to be 18,907, based on the lesser value of the upper limits of precision and linearity. The LLOQ of both assays will be further evaluated using additional samples at the lower end of the titer range during assay validation.

#### 3.1.4. Specificity

For the PRN+ SBA, for all five samples, the inhibition observed was ≥75% for the *B. pertussis* WCE, the homologous competitor, and ≤50% for diphtheria toxoid, the heterologous competitor (Supplementary Table S3). Similarly, for the PRN− SBA, for all five samples, the inhibition observed was ≥75% for the WCE, and ≤50% for the diphtheria and tetanus toxoids in ≥80% of samples (Supplementary Table S4). Thus, the descriptive target criteria were reached for specificity in both PRN+ and PRN− SBAs in human serum samples, demonstrating that both assays are specific to *B. pertussis*, and not affected by heterologous competition.

#### 3.1.5. Matrix Interference

For both the PRN+ and PRN− SBAs, the presence of hemoglobin, bilirubin, or lipid at high levels in serum samples did not interfere with the SBA for *B. pertussis* PRN+ B1917, confirming the lack of matrix interference of this assay. For all samples spiked with each interferent, the recovery was within 50% and 200%, and the negative sample remained negative (Supplementary Tables S5 and S6).

#### 3.1.6. Freeze–Thaw Assessment

For the PRN+ SBA, the GMRs of the bench top condition (up to 24 h and 34 min), and of five and 10 freeze/thaw cycle conditions in comparison to the reference condition were 0.95, 1.15 and 1.04, respectively (Supplementary Table S7). For the PRN− SBA, the GMRs of the bench top condition (up to 24 h), and of five and 10 freeze/thaw cycle conditions in comparison to the reference condition were 0.99, 1.15 and 1.16, respectively (Supplementary Table S8). These values were within the pre-defined target acceptance criterion of 0.80 to 1.25 and were considered acceptable, indicating bench top stability time of at least 24 h and that samples can undergo up to 10 freeze thaws without affecting results, enabling repeat analyses if required.

#### 3.1.7. Robustness

**Figure 6:**
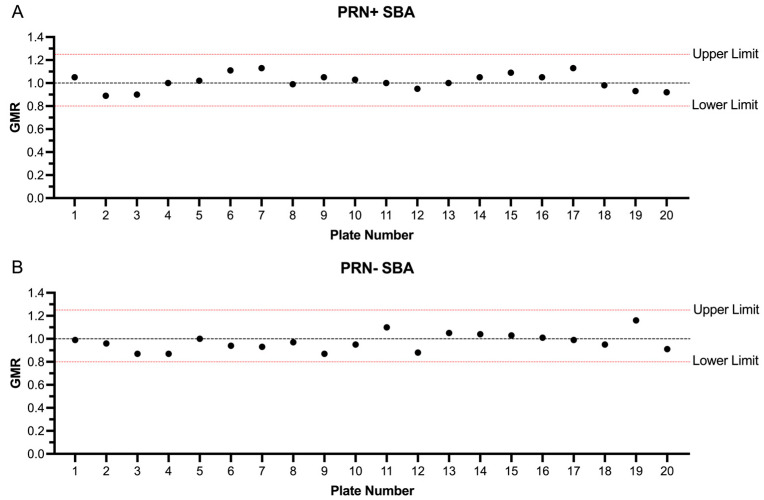
Robustness for (**A**) PRN+ SBA and (**B**) PRN− SBA. Assessment plotting the GMR of SBA titers for 6 samples on each plate over 20 runs to their reference titers (the GMT of the last 5 plates), represented by black dots. All GMRs for both assays were within the target acceptance criteria limits of 0.8 to 1.25.

For both the PRN+ and PRN− SBAs, when assessing the maximum plates per run, the plate GMRs were between 0.80 and 1.25 for all samples tested, supporting a maximum plate run of 120 samples prepared on 20 plates with six samples each (Figure 6). Additionally, for the extended stability test, all GMRs were within a range of 0.94 to 1.15 after 10 days of incubation at 4 °C, enabling flexibility during analysis of large numbers of samples (Supplementary Table S9).

#### 3.1.8. Quality Control Range Determination

**Figure 7:**
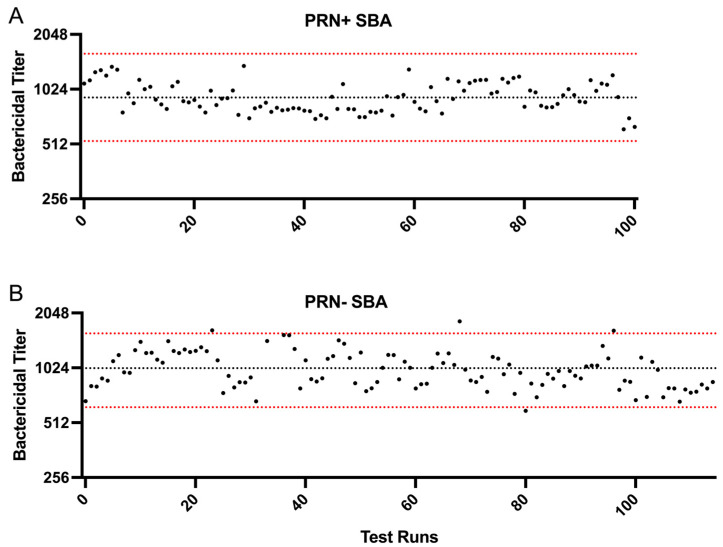
Quality control range determination and trend over time for (**A**) PRN+ SBA and (**B**) PRN− SBA. For PRN+ SBA, GMT (black dotted line, titer = 921) and upper and lower limits (red dashed lines, titers 1602 and 531 respectively). For PRN− SBA, GMT (black dotted line, titer = 991) and upper and lower limits (red dashed lines, titers 1581 and 622 respectively).

For the PRN+ SBA, the mid-positive QC sample was tested 101 times by two analysts over 3 weeks and 12 separate assay runs. The GMT of the sample set was 921, with lower and upper range limits determined to be 531 and 1602, respectively (Figure 7A). Additionally, the negative control was negative in 100 out of 101 instances, deeming it fit to use as a negative control for plate acceptance criteria during clinical analyses.

For the PRN− SBA, the mid-positive QC sample was tested 115 times by three analysts over 5 weeks and 14 separate assay runs. The GMT of the sample set was 991, with lower and upper range limits determined to be 622 and 1581 respectively (Figure 7B). Additionally, the negative control was negative 100% of the time (115 out of 115 instances), and was also incorporated as a plate acceptance criteria.

#### 3.1.9. Bacteria Control with Active Human Complement Range Determination

**Figure 8:**
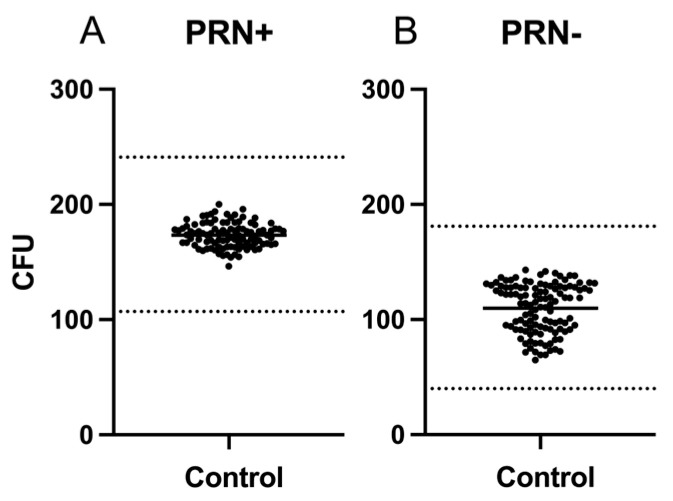
CFU count for the bacteria with active complement control for (**A**) PRN+ SBA and (**B**) PRN− SBA. Black dotted lines represent the upper and lower range for each assay. Solid black lines represent mean CFU count.

For the PRN+ SBA, this control was evaluated by analyzing 807 data points (column 11, 8 wells per plate) generated by two analysts conducting 13 runs over 6 weeks. For the PRN− SBA, 918 datapoints were reported by three analysts conducting 15 runs over 5 weeks. For the PRN+ and PRN− SBAs, the CFU values were distributed from the average CFU with CVs of 9.7 and 21.2, respectively. For the PRN+ SBA, the mean CFU was determined to be 174 with an SD of 17. The range was determined to be 107 CFUs to 241 CFUs (Figure 8A). For the PRN− SBA, the mean CFUs was determined to be 110 with an SD of 23. Thus, the range was determined to be 40 CFUs to 181 CFUs (Figure 8B).

### 3.2. Analysis of Clinical Samples from Phase 2B Clinical Study

**Figure 9:**
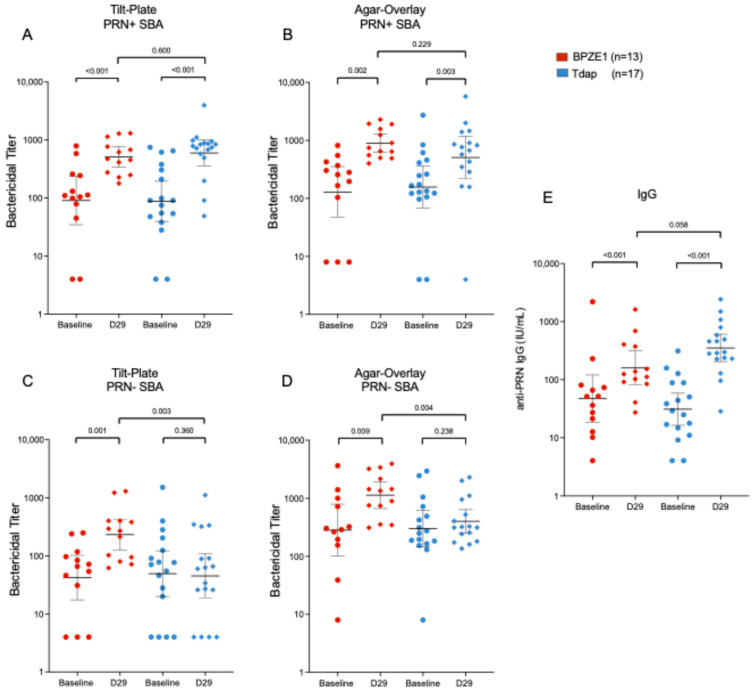
Serum bactericidal titers (**A**–**D**) and anti-PRN IgG (**E**) at baseline (circles) and day 29 (diamonds) post-vaccination against PRN+ *B. pertussis* (**A**,**B**) and PRN− *B. pertussis* (**C**,**D**), utilizing the reference tilt-plate method (**A**,**C**), or the new high-throughput agar-overlay SBA (**B**,**D**). GMT ± 95% CIs; *p*-values were calculated with paired or independent *t*-test on a logarithmic scale. D29 = day 29; Tdap = tetanus–diphtheria–acellular pertussis vaccine. Values less than the LLOQ were interpreted as LLOQ for each assay. Panels (**A**,**C**) reproduced from [27]. BPZE1 (n = 13) is red, Tdap (n = 17) is blue. IU, international units.

**Figure 10:**
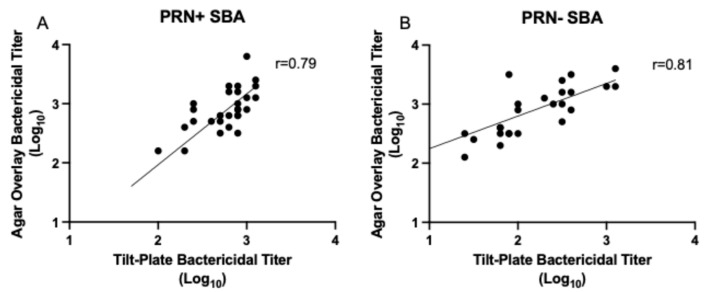
Correlation of serum bactericidal titers from the tilt-plate method (x-axis) and the high-throughput agar-overlay method (y-axis) for (**A**) PRN+ SBA and (**B**) PRN− SBA. Thirty samples were analyzed from a Phase 2b clinical trial with 13 subjects receiving BPZE1 and 17 received Tdap. Black dots represent individual sample titers. Black line is the linear regression.

Previously published bactericidal titers obtained using the tilt-plate method from 30 participants from a Phase 2b clinical trial of BPZE1 (Figure 9A,C) were compared with titers obtained using the new agar-overlay method (Figure 9B,D). Both methods show concordant results. Additionally, baseline GMTs for both PRN+ and PRN− SBAs were similar between the vaccine groups within in each assay. With the PRN+ SBA, BPZE1 and Tdap induced similar titers on day 29 post-vaccination despite Tdap participants having higher anti-PRN IgG concentrations (Figure 9E), demonstrating that BPZE1 induces more bactericidal antibodies than are represented solely by anti-PRN IgG. This is further reinforced because only BPZE1 induced bactericidal antibodies against PRN− *B. pertussis*, showing a difference in GMT at day 29 post-vaccination compared with baseline GMTs (*p* = 0.009), while Tdap did not induce an increase at day 29 compared to baseline (*p* = 0.238). Additionally, when comparing bactericidal titers from the same samples between the tilt-plate and high-throughput agar-overlay methods, a strong correlation was observed for both PRN+ and PRN− SBAs, with Pearson’s r values of 0.81 and 0.79 respectively (Figure 10), further supporting the concordance of the assay methods.

## 4. Discussion

Vaccine pressure from anti-PRN antibodies induced by currently approved aPVs has resulted in higher incidences of *B. pertussis* infections from PRN− *B. pertussis* strains [16,17]. In fact, the majority of circulating strains in many countries using aPVs prior to the COVID-19 pandemic were PRN− strains [14,16,32,33]. Despite recent increases in PRN+ *B. pertussis* strains after the COVID-19 pandemic, suggested to be caused by reduced boosting of anti-PRN immunity following circulation of PRN− strains during the pandemic combined with importation of PRN+ strains from WPV countries [34], PRN− strains still continue to circulate [4,35]. Therefore, utilizing SBAs with PRN+ and PRN− strains remain of importance when developing new pertussis vaccines to illustrate breadth of bactericidal capability.

The tilt-plate SBA for *B. pertussis* was initially developed to support research questions and early-phase clinical development. The method has proven to be accurate and reproducible. One advantage of the tilt-plate assay is that it can be run without the need for automated equipment, as each sample is manually plated onto standard 100 mm agar plates. While this is feasible for studies supporting research questions and early-phase clinical development, the resources add up quickly, considering raw material use, incubator space, as well as the time to process many samples. Thus, a need remained for a method that could support the larger studies required for late-stage clinical development with the evaluation of many hundreds if not thousands of samples in a relatively compressed timeframe. Additional considerations for a more streamlined method include practical handling and disposal of thousands of agar plates. By removing the secondary plating step, using the agar-overlay method, large volumes of reagents are spared, and environmental, health and safety resources are reduced substantially as well.

In this study, we successfully developed high-throughput agar-overlay SBAs against both PRN+ and PRN− *B. pertussis* that are precise, accurate, linear, specific, robust, and stable, which will enable further characterization of the functional immune response induced by pertussis vaccines, including BPZE1. To develop these assays, IgG- and IgM-depleted human serum was combined with heat-treated serum samples and bacteria to induce antibody and complement-dependent bactericidal activity. Others have previously demonstrated bactericidal activity against *B. pertussis* using guinea pig [36] or pre-colostral calf serum [37] as complement sources. However, utilizing human complement provides a direct link to the relevant target population for pertussis vaccines and has been demonstrated as a preferred source for other bacterial vaccines, such as those targeting *Neisseria meningitidis* [38,39].

While assessing precision, it is worth noting that for the PRN+ assay, the LLP was established using Sample 2, which had 5 of 12 reportable titers, not 6 of 12 as specified a priori as a target criterion. We found this to be acceptable for this stage of development; however, the LLP will be fully established during method validation (before Phase 3 clinical analysis) where we will use a larger panel of samples, including multiple samples with low titers.

Initially, we attempted to develop the high-throughput SBAs using the Tohama-1 streptomycin- and nalidixic acid-resistant derivative strain BPSM [40], and a PRN− derivative of BPSM [41], since BPSM is the parental strain of the live attenuated vaccine BPZE1. Although the Tohama-1 strain had previously been investigated along with other *B. pertussis* strains for interactions with human complement [42,43], and was shown to be sensitive to non-specific, antibody-independent killing mediated by complement, the behavior of the BPSM strains in the agar-overlay SBA was unknown. After extensive testing, BPSM was found to be too susceptible to non-specific complement-mediated killing, with up to 80% killing observed when using only 2% complement. We therefore evaluated *B. pertussis* strain B1917, since it was more representative of current isolates. In addition, we have previously shown bactericidal activity from serum samples collected during a BPZE1 Phase 2b study on both PRN+ and PRN− B1917 strains using the tilt-plate SBA method [27]. Furthermore, B1917 was selected for use in human challenge studies to investigate colonization of virulent *B. pertussis* [44,45] and to test the protective efficacy of BPZE1 [23], where a 98.7% reduction in bacterial burden in BPZE1 vaccinees was demonstrated compared to placebo controls.

While BPZE1 protected mice against *B. pertussis* strains, including antibiotic-resistant PRN− variants [46], a human challenge model for PRN− *B. pertussis* has not yet been established. Thus, SBAs utilizing human serum and complement serve as a valuable proxy to demonstrate the induction of potentially protective antibodies for vaccine candidates. Using the tilt-plate pertussis SBA, sera from convalescent pertussis patients successfully killed multiple knock-out strains, including PRN− B1917, demonstrating a broad repertoire of bactericidal antibodies, whereas following aP vaccination only PRN induced bactericidal antibodies [15]. Like wild-type *B. pertussis*, the live attenuated BPZE1 vaccine also contains thousands of proteins, induces broad humoral and cellular immune responses including serum bactericidal activity against both PRN+ and PRN− strains, and provides comprehensive protection in animal models. These combined findings suggest that BPZE1 will effectively protect humans against diverse *B. pertussis* strains as well.

When comparing results of test samples on the new high-throughput agar-overlay SBAs and the established tilt-plate SBAs, both assays correlated well to one another with regard to bactericidal titer, showing Pearson’s r of ~0.80 between the assays. Similar observations were made between the two types of assays when serum from BPZE1 vaccinees was used. Both assays demonstrated BPZE1-induced bactericidal activity to both PRN+ and PRN− *B. pertussis,* while serum from Tdap vaccinees only induced bactericidal activity to PRN+ *B. pertussis*. This gives further confidence that the antibody-dependent complement-mediated killing mechanisms performed consistently between the assay methods. It is worth noting that the tilt-plate and agar-overlay SBAs differ with respect to the agar type, method of incubation, complement source (prepared in-house [47] vs. commercially), and the numbers of bacteria added to each well, likely accounting for numeric differences in titers between the assays. Despite these differences, the SBA methods yielded comparable results. Therefore, we conclude that both PRN+ and PRN− high-throughput assays are suitable for their intended use, i.e., to measure functional serum antibody responses in clinical studies of BPZE1 and other pertussis vaccines.

## 5. Conclusions

In this study we describe the development and qualification of SBAs capable of testing hundreds of clinical samples per day using a semi-automated high-throughput assay. For Phase 3 studies, the newly developed agar-overlay SBAs will undergo validation, repeating similar studies with a larger number of samples across the range of expected titers, increasing replicates, analysts, and days of testing to build a robust dataset ensuring accurate, reliable, and reproducible data compliant with global regulatory agency requirements, to further demonstrate broad functional immune responses induced by vaccination with BPZE1.

## Figures and Tables

**Table 1:** Precision analysis of PRN+ and PRN− SBA.

Assay	N *	CV Intra-Assay Precision (%)	CV Intermediate Precision (%)
PRN+ SBA	193	10.8	25.5
PRN− SBA	216	19.3	28.4

* N represents the number of evaluable samples used in the precision analysis for each assay.

## Data Availability

The original contributions presented in this study are included in the article/Supplementary Materials. Further inquiries can be directed to the corresponding author.

## Supplementary Materials

The following supporting information can be downloaded at: https://www.mdpi.com/article/10.3390/vaccines14060492/s1, Table S1: Precision assessment of PRN+ SBA; Table S2: Precision assessment of PRN− SBA; Table S3: Specificity assessment of PRN+ SBA; Table S4: Specificity assessment of PRN− SBA; Table S5: Matrix interference for PRN+ SBA; Table S6: Matrix interference for PRN− SBA; Table S7: Freeze–thaw analysis of PRN+ SBA; Table S8 Freeze–thaw analysis of PRN− SBA; Table S9: Extended reading time for PRN+ SBA; Table S10: Extended reading time for PRN− SBA.

## Abbreviations

The following abbreviations are used in this manuscript:

aP	Acellular pertussis
aPV	Acellular pertussis vaccine
ANOVA	Analysis of variance
B1917	*Bordetella pertussis* strain B1917
BG	Bordet–Gengou
BPSM	*Bordetella pertussis* streptomycin- and nalidixic acid-resistant derivative of Tohama-1
BPZE1	Live attenuated intranasal *Bordetella pertussis* vaccine candidate
BSA	Bovine serum albumin
C’	Complement
CFU	Colony-forming unit
CI	Confidence interval
CLSI	Clinical and Laboratory Standards Institute
CO_2_	Carbon dioxide
CV	Coefficient of variation
D29	Day 29 post-vaccination
ECL	Electrochemiluminescence
FHA	Filamentous hemagglutinin
FIM	Fimbriae
GCLP	Good Clinical Laboratory Practice
GMT	Geometric mean titer
GMR	Geometric mean ratio
HBSS	Hanks’ balanced salt solution
ICH	International Conference for Harmonisation
IgG	Immunoglobulin G
IgM	Immunoglobulin M
IU	International unit
LLOQ	Lower limit of quantitation
LLL	Lower limit of linearity
LLP	Lower limit of precision
MAC	Membrane attack complex
NIBSC	National Institute for Biological Standards and Control
PRN	Pertactin
PRN+	Pertactin-positive
PRN−	Pertactin-negative
QC	Quality control
R^2^	Coefficient of determination
RIVM	National Institute for Public Health and the Environment (The Netherlands)
RT	Room temperature
SBA	Serum bactericidal assay
SD	Standard deviation
SI	Supplementary Information
Tdap	Tetanus–diphtheria–acellular pertussis vaccine
ULOQ	Upper limit of quantitation
ULL	Upper limit of linearity
ULP	Upper limit of precision
WCE	Whole-cell extract
wPV	Whole-cell pertussis vaccine
WHO	World Health Organization
