# Supplementary material for: Development of High-Throughput Serum Bactericidal Assays for Bordetella pertussis to Evaluate BPZE1
Source: Vaccines (Basel). 2026 May 30;14(6):492. doi: 10.3390/vaccines14060492 (PMC13307608; doi:10.3390/vaccines14060492)
Supplement: Supplementary file 1 [file vaccines-14-00492-s001.zip › vaccines-4310548-supplementary.pdf]

Supplementary Information

SI Table 1

Table S1. Precision assessment of PRN+ SBA

Sample	Starting Dilution in Plate	Number of Valid Values (N)	Geometric Mean Titer (SBA Titer)	CV (%)
1	8	0	<8[a]	NA
2	8	5[b]	9	11.20
3	8	1	NA[a]	NA
4	32	12	97	37.15
5	32	11	247	53.50
6	32	12	228	29.24
7	32	12	351	34.27
8	32	4	NA[a]	NA
9	32	12	430	30.92
10	32	12	475	18.62
11	8	12	820	20.49
12	32	11	894	20.66
13	32	12	651	11.22
14	32	12	881	10.57
15	32	12	1090	21.09
16	32	12	1729	11.78
17	32	10	2155	18.15
18	512	12	1960	17.51
19	512	12	9863	26.01
20	512	12	20515	25.18

[a] GMT and CV not considered for analysis as less than 6 of the 12 results that are reportable (and positive).

[b] Due to this being an early-phase assay qualification study, sample 2 was considered for the analysis, even though only 5 values were valid. Abbreviations: Geometric mean titer (GMT); coefficient of variation (CV); Serum bactericidal assay (SBA)

Table S2

Table S2 Precision assessment of PRN- SBA

Sample	Starting Dilution in Plate	Number of Valid Values (N)	GMT (SBA Titer)	CV (%)
1	8	0	<8[a]	NA[a]
2	8	12	952	18.21
3	8	11	107	44.40
4	8	11	20	36.65
5	8	12	38	33.05
6	8 and 16[b]	10	77	14.67
7	32	11	146	28.68
8	32	11	625	26.57
9	32	12	2160	29.72
10	32	12	2004	23.67
11	32	12	1707	40.24
12	32	11	1714	10.70
13	32	12	367	26.45
14	32	11	280	33.14
15	32	11	945	13.13
16	32	12	637	25.14
17	32	12	184	17.54
18	512	11	19387	15.37
19	512	11	3373	16.76
20	512	11	8136	31.66

[a] GMT and CV not considered for analysis as less than 6 of the 12 results that are reportable (and positive).

[b] Starting dilution in plate of Sample 6 was 8 for precision day 1 and changed to 16 for precision days 2 and 3 to avoid a hook effect detected in precision day 1.

Table S3

Specificity assessment of PRN+ SBA using homologous and heterologous competitors

Sample	Inhibitor	Concentration of Inhibitor (µg/mL)	SBA Titer	%Inhibition
**Sample 6**	No inhibitor	NA	277	NA
	Bordetella pertussis Tohama I WCE	25.0	32	88
	Diphtheria Toxoid	25.0	138	50
**Sample 7**	No inhibitor	NA	348	NA
	Bordetella pertussis Tohama I WCE	25.0	32	91
	Diphtheria Toxoid	25.0	273	22
	No inhibitor	NA	494	NA

**Sample 8**	*Bordetella pertussis* Tohama I WCE	25.0	32	94
	Diphtheria Toxoid	25.0	541	-10
**Sample 9**	No inhibitor	NA	1577	NA
	Bordetella pertussis Tohama I WCE	25.0	32	98
	Diphtheria Toxoid	25.0	891	44
**Sample 10**	No inhibitor	NA	6889	NA
	Bordetella pertussis Tohama I WCE	25.0	512	93
	Diphtheria Toxoid	25.0	5076	26

Table S4

Specificity assessment of PRN- SBA using homologous and heterologous competitors

**Sample**	**Competitor**	**Concentration of competitor (µg/mL)**	**SBA Titer**	**% Inhibition**
**Sample 1**	No competitor	NA	143	NA
	Bordetella pertussis Tohama I WCE	25.0	32	78
	Diphtheria Toxoid	25.0	134	6
	Tetanus Toxoid	25.0	147	-3
**Sample 2**	No competitor	NA	300	NA
	Bordetella pertussis Tohama I WCE	25.0	32	89
	Diphtheria Toxoid	25.0	213	29
	Tetanus Toxoid	25.0	64	79
**Sample 3**	No competitor	NA	432	NA
	Bordetella pertussis Tohama I WCE	25.0	32	93
	Diphtheria Toxoid	25.0	500	-16
	Tetanus Toxoid	25.0	612	-42
**Sample 4**	No competitor	NA	770	NA
	Bordetella pertussis Tohama I WCE	25.0	32	96
	Diphtheria Toxoid	25.0	659	14
	Tetanus Toxoid	25.0	494	36
**Sample 5**	No competitor	NA	6766	NA
	Bordetella pertussis Tohama I WCE	25.0	512	92
	Diphtheria Toxoid	25.0	5613	17
	Tetanus Toxoid	25.0	8192	-21

[a] SBA titer results of <32 and <512 were expressed as "32" and "512" respectively for calculation purposes. The corresponding % inhibition is a minimum.

Table S5. Matrix Interference for PRN+ SBA

Sample	Level of Interferent	Type of Interferent	SBA Titer	%Recovery
**A**	NA	No interferent	<8	NA
	3+	Hemoglobin	<8	NA
	3+	Bilirubin	<8	NA
	3+	Lipid	<8	NA
**B**	NA	No interferent	177	100
	3+	Hemoglobin	220	124
	3+	Bilirubin	209	118
	3+	Lipid	174	98
**C**	NA	No interferent	244	100
	3+	Hemoglobin	262	107
	3+	Bilirubin	219	90
	3+	Lipid	274	112
**D**	NA	No interferent	852	100
	3+	Hemoglobin	737	86
	3+	Bilirubin	724	85
	3+	Lipid	708	83
**E**	NA	No interferent	1065	100
	3+	Hemoglobin	1040	98
	3+	Bilirubin	957	90
	3+	Lipid	875	82
**F**	NA	No interferent	13674	100
	3+	Hemoglobin	12062	88
	3+	Bilirubin	9842	72
	3+	Lipid	9368	69

SI Table 6. Matrix Interference for PRN- SBA Assay

Sample	Level of Interferent	Type of Interferent	SBA Titer	% Recovery
**A**	NA	No interferent	<8	NA
	3+	Hemoglobin	<8	NA
	3+	Bilirubin	<8	NA
	3+	Lipid	<8	NA

**B**	NA	No interferent	147	100
	3+	Hemoglobin	70	48
	3+	Bilirubin	66	45
	3+	Lipid	110	75
**C**	NA	No interferent	281	100
	3+	Hemoglobin	311	111
	3+	Bilirubin	242	86
	3+	Lipid	259	92
**D**	NA	No interferent	761	100
	3+	Hemoglobin	651	86
	3+	Bilirubin	773	102
	3+	Lipid	664	87
**E**	NA	No interferent	1586	100
	3+	Hemoglobin	1817	115
	3+	Bilirubin	1609	101
	3+	Lipid	1476	93
**F**	NA	No interferent	6421	100
	3+	Hemoglobin	5793	90
	3+	Bilirubin	5736	89
	3+	Lipid	5676	88

Table S7. Freeze-thaw Analysis of PRN+ SBA Assay

Sample	Ratios (24 hours and 34 minutes bench top/Ref)	Ratios (5 FT/Reference)	Ratios (10 FT/Reference)
**G**	0.76	1.04	0.71
**H**	0.91	0.97	0.88
**I**	1.25	1.34	1.41
**J**	0.93	1.13	1.09
**K**	1.15	1.34	1.10
**L**	0.77	1.13	1.18
**GMR**	0.95	1.15	1.04

SI Table 8. Freeze-thaw Analysis of PRN- SBA Assay

Sample	Ratios (24 hours bench top/Reference)	Ratios (5 FT/Reference)	Ratios (10 FT/Reference)
1	NA	NA	NA
2	0.92	1.02	0.91
3	0.95	0.80	0.86
4	1.06	1.31	1.28
5	0.90	1.31	1.35
6	1.10	1.44	1.51
GMR:	0.99	1.15	1.16

SI Table 9. Extended Reading Time for PRN+ SBA Assay

GMR of Extended Reading Condition vs Reference Condition

Sample	Replicate ID	Ratio to the Reference			
		GMR after 6 Days	GMR after 10 Days	GMR after 6 Days	GMR after 10 Days
		Analyst 1		Analyst 2	
1	1	1.00	1.00	1.00	1.00
2	1	1.00	1.00	1.00	1.00
3	1	Not Applicable	Not Applicable	Not Applicable	Not Applicable
4	1	0.99	1.00	1.00	1.03
5	1	1.01	1.01	0.98	1.04
6	1	0.99	0.99	0.99	0.99
7	1	0.98	0.98	1.06	1.07
8	1	Not Applicable	Not Applicable	Not Applicable	Not Applicable
9	1	0.98	0.98	0.99	0.99
10	1	1.00	1.01	1.02	1.03
11	1	0.99	0.99	0.99	1.02
12	1	1.01	1.01	0.99	1.02
13	1	0.98	0.98	0.98	1.01
14	1	1.01	1.00	1.00	1.00
15	1	1.03	1.00	0.99	1.00
16	1	0.98	0.98	1.00	1.01

17	1	0.97	0.97	Not Applicable	Not Applicable
18	1	0.98	1.00	0.97	0.92
19	1	0.96	0.98	1.02	0.96
20	1	1.01	1.00	1.02	1.06
1	2	1.00	1.00	1.00	1.00
2	2	1.00	1.00	1.13	1.00
3	2	Not Applicable	Not Applicable	Not Applicable	Not Applicable
4	2	0.99	0.99	1.01	1.04
5	2	1.00	1.00	1.00	1.00
6	2	1.00	1.00	0.99	1.01
7	2	0.96	0.98	1.00	1.03
8	2	Not Applicable	Not Applicable	Not Applicable	Not Applicable
9	2	0.99	1.02	0.99	1.01
10	2	0.99	1.01	1.00	0.98
11	2	0.99	0.96	0.94	0.96
12	2	0.98	0.98	Not Applicable	Not Applicable
13	2	1.02	1.02	0.97	1.03
14	2	1.00	1.00	0.98	1.00
15	2	0.97	0.99	0.98	0.97
16	2	1.00	1.02	1.00	1.01
17	2	0.96	0.98	Not Applicable	Not Applicable
18	2	1.00	1.00	0.98	0.99
19	2	0.98	1.00	0.98	0.92
20	2	0.99	1.02	1.05	0.98
Samples having no SBA Titers (Invalid Result):		**4**	**4**	**7**	**7**
# of samples having valid results		**36**	**36**	**33**	**33**
Total number of samples with Ratio between 0.80 and 1.25:		**36**	**36**	**33**	**33**
Percentage of samples with Ratio between 0.80 and 1.25:		**100%**	**100%**	**100%**	**100%**

SI Table 10. Extended Reading Time for PRN- SBA Assay

**Sample**	**Replicate**	**SBA Titer**			**Ratio to the Reference**	
		**Reference (T0)**	**Reading +6**	**Reading +10**	**Reading +6 / Ref**	**Reading +10 / Ref**
1[a]	1	8	8	8	1.00	1.00
2	1	1137	1137	1145	1.00	1.01
3	1	256	271	272	1.06	1.06
4	1	22	22	23	1.02	1.03
5	1	28	29	31	1.05	1.10
6	1	80	79	77	0.99	0.96

7	1	153	161	159	1.05	1.04
8	1	840	834	854	0.99	1.02
9	1	3069	3104	3123	1.01	1.02
10	1	2318	2308	2329	1.00	1.00
11	1	2451	2435	2464	0.99	1.01
12	1	2435	2511	2580	1.03	1.06
13	1	414	417	425	1.01	1.03
14	1	250	362	406	1.45	1.62
15	1	To Dilute	To Dilute	To Dilute	Not Applicable	Not Applicable
16	1	835	818	861	0.98	1.03
17	1	164	164	166	1.00	1.01
18	1	18390	18820	19349	1.02	1.05
19	1	3808	4096	4266	1.08	1.12
20	1	11585	11585	12040	1.00	1.04
1[a]	2	8	8	8	1.00	1.00
2	2	927	912	934	0.98	1.01
3	2	99	99	103	1.00	1.04
4	2	Invalid Result	Invalid Result	Invalid Result	Not Applicable	Not Applicable
5	2	27	26	27	0.96	1.02
6	2	67	68	66	1.02	0.99
7	2	150	143	143	0.96	0.95
8	2	664	670	673	1.01	1.01
9	2	2956	3158	3198	1.07	1.08
10	2	2385	2393	2332	1.00	0.98
11	2	1722	1815	1773	1.05	1.03
12	2	1801	1833	1833	1.02	1.02
13	2	406	422	414	1.04	1.02
14	2	351	388	362	1.11	1.03
15	2	951	953	1076	1.00	0.99
16	2	607	611	684	1.01	1.01
17	2	177	175	200	0.99	1.02
18	2	22604	23170	26008	1.03	1.15
19	2	4891	5027	5043	1.03	1.03
20	2	8192	7938	7692	0.97	0.94
				**Samples having no SBA Titers (Invalid Result / To Dilute):**	2	2
				**# of samples having valid results:**	38	38
				**Total number of samples with Ratio between 0.80 and 1.25:**	37	37
				**Percentage of samples with Ratio between 0.80 and 1.25:**	97%	97%

Note: [a]: SBA titers < 8 results were replaced with SBA Titers of 8 for calculation purposes.
